# PGE2/EP4 skeleton interoception activity reduces vertebral endplate porosity and spinal pain with low-dose celecoxib

**DOI:** 10.1038/s41413-021-00155-z

**Published:** 2021-08-02

**Authors:** Peng Xue, Shenyu Wang, Xiao Lyu, Mei Wan, Xialin Li, Lei Ma, Neil C. Ford, Yukun Li, Yun Guan, Wenyuan Ding, Xu Cao

**Affiliations:** 1 Department of Orthopaedic Surgery, The Johns Hopkins University School of Medicine, Baltimore, MD USA; 2 Department of Endocrinology, The Third Hospital of Hebei Medical University, Shijiazhuang, Hebei P. R. China; 3 Key Laboratory of Orthopaedic Biomechanics of Hebei Province, Shijiazhuang, Hebei P.R. China; 4 Department of Spine Surgery, The Third Hospital of Hebei Medical University, Shijiazhuang, Hebei P. R. China; 5 Department of Anesthesiology and Critical Care Medicine, The Johns Hopkins University School of Medicine, Baltimore, MD USA

**Keywords:** Diseases, Bone

## Abstract

Skeletal interoception regulates bone homeostasis through the prostaglandin E2 (PGE2) concentration in bone. Vertebral endplates undergo ossification and become highly porous during intervertebral disc degeneration and aging. We found that the PGE2 concentration was elevated in porous endplates to generate spinal pain. Importantly, treatment with a high-dose cyclooxygenase 2 inhibitor (celecoxib, 80 mg·kg^−1^ per day) decreased the prostaglandin E2 concentration and attenuated spinal pain in mice with lumbar spine instability. However, this treatment impaired bone formation in porous endplates, and spinal pain recurred after discontinuing the treatment. Interestingly, low-dose celecoxib (20 mg·kg^−1^ per day, which is equivalent to one-quarter of the clinical maximum dosage) induced a latent inhibition of spinal pain at 3 weeks post-treatment, which persisted even after discontinuing treatment. Furthermore, when the prostaglandin E2 concentration was maintained at the physiological level with low-dose celecoxib, endplate porosity was reduced significantly, which was associated with decreased sensory nerve innervation and spinal pain. These findings suggest that low-dose celecoxib may help to maintain skeletal interoception and decrease vertebral endplate porosity, thereby reducing sensory innervation and spinal pain in mice.

## Introduction

Low back pain (LBP) is a common disease leading to a decline in mobility and to frailty worldwide.^1,2^ LBP is also the leading cause of activity limitations and work absences, affecting 80% of people at some point during their lives.^3^ The economic burden associated with LBP exceeds $90 billion per year in the United States.^4^ Pain, particularly LBP, is the most prominent symptom of skeletal degeneration and can be chronic or episodic.^5^ Pain is a major reason people seek medical attention^6^, and persistent pain at rest profoundly affects quality of life,^7,8^ especially in the elderly population, for whom it often leads to functional decline.^9^ Unfortunately, we still do not understand the cause of LBP, and no effective disease-modifying therapy exists.

Using magnetic resonance imaging, researchers have found signal changes in the vertebral endplate in patients with LBP.^10–12^ Specifically, the size of the area showing Modic changes or endplate defects is strongly associated with LBP.^13–15^ Endplates undergo ossification and become highly porous during intervertebral disc degeneration.^16–18^ It has also been reported that more nerve innervation occurs in porous endplates than in normal endplates or in the degenerative nucleus pulposus.^19^ We recently found^20^ that osteoclasts generated porous endplates with sensory innervation in aged mice and mice with lumbar spine instability (LSI) and that elevated concentrations of prostaglandin E2 (PGE2) in porous endplates activate sensory nerves, causing spinal pain. Importantly, blocking PGE2/PGE2 receptor 4 (EP4) signaling ameliorated spinal pain behavior in EP4 knockout mice.^20^ Focal inflamed tissue releases PGE2, which acts as a neuromodulator to increase neuronal excitability.^21–23^ PGE2 generates inflammatory hypersensitivity and sensory neuron sensitization via its receptor EP4.^24,25^ Moreover, the PGE2 signaling pathway can activate different pain-related ion channels, such as the induction of calcium or sodium influx through transient receptor potential vanilloid 1 (TRPV1)^26^ or the Na_v_1.8 channel.^20^

We have identified PGE2/EP4-mediated skeletal interoception, in which PGE2 secreted by osteoblasts activates EP4 in sensory nerves to induce phosphorylation of cAMP-response element binding protein (CREB) in the hypothalamus as an upstream interoceptive signaling pathway, where sympathetic activity is decreased to promote osteoblastic bone formation as the downstream interoceptive action.^27^ The skeleton has abundant sensory and sympathetic innervations,^28^ and its homeostasis is regulated by this skeletal interoception.^27,29^ Sensory nerves sense changes in bone density, mechanical stress, and metabolic activity to control bone resorption and formation through skeletal interoception. Specific deletion of sensory nerves in bone impairs bone mass accrual.^30^ Mechanical stress and bone density regulate the secretion of PGE2 by osteoblasts to decrease sympathetic activity, which promotes bone formation. High sympathetic tone increases catabolic activity in bone via CREB and the serotonin signaling pathway in the hypothalamus.^27,31,32^ Importantly, in osteoarthritis and spinal degeneration conditions, the PGE2 concentration is high in porous subchondral bone and endplates, which generates pain. Indeed, antagonists of the EP4 receptor reduced acute or chronic pain, such as osteoarthritis-related pain.^33,34^ Physiological levels of PGE2 maintain appropriate skeletal interoception activity to maintain bone homeostasis, whereas higher PGE2 concentrations induce pain to protect against injury or fracture due to low bone density in skeletal diseases, such as spine degeneration, osteoarthritis, and osteoporosis. Thus, maintaining the physiological level of PGE2 could modify the disease and reduce pain through skeletal interoception.

Nonsteroidal anti-inflammatory drugs (NSAIDs) are currently recommended as a first-line treatment for LBP.^35^ As a major class of drugs used to treat chronic pain, NSAIDs reduce the production of PGE2, suggesting the importance of understanding the sensitizing effects of prostaglandins on sensory nerve and nociceptive dorsal root ganglion (DRG) neurons. NSAIDs comprise 2 types: nonselective NSAIDs (e.g., ibuprofen) that inhibit both cyclooxygenase 1 (COX-1) and cyclooxygenase 2 (COX-2) enzymes and selective NSAIDs (e.g., celecoxib) that inhibit only the COX-2 enzyme. Selective NSAIDs were developed since nonselective NSAIDs might lead to adverse gastrointestinal effects.^36,37^ Selective COX-2 inhibitors decrease the risk of gastrointestinal complications^38^ but may increase the risk of cardiovascular adverse events.^39,40^ Rofecoxib was withdrawn from the market for this reason.^41–43^ Evidence shows that these risks are duration- and dose-dependent.^44,45^ Therefore, when NSAIDs are prescribed, they should be prescribed at the lowest effective dose for the shortest duration.^46^ COX-2 inhibitors are widely used for skeletal pain treatment, with annual sales totaling more than $7 billion in the U.S. Clinical trials have shown that a high dosage of COX-2 inhibitors is necessary for pain relief.^47,48^ For celecoxib, the typical dosage is 200–400 mg·d^−1^.

In this study, we investigated whether treatment with a low-dose COX-2 inhibitor maintains appropriate PGE2/EP4-mediated skeletal interoception-induced activity, thereby decreasing vertebral endplate porosity and LBP. Our results showed that low-dose celecoxib (20 mg·kg^−1^ per day, which is equivalent to one-quarter of the clinical maximum dosage) decreased endplate porosity and reduced sensory innervation and spinal pain hypersensitivity.

## Results

### Low-dose celecoxib reduced spinal pain hypersensitivity and endplate porosity

**Fig. 1:**
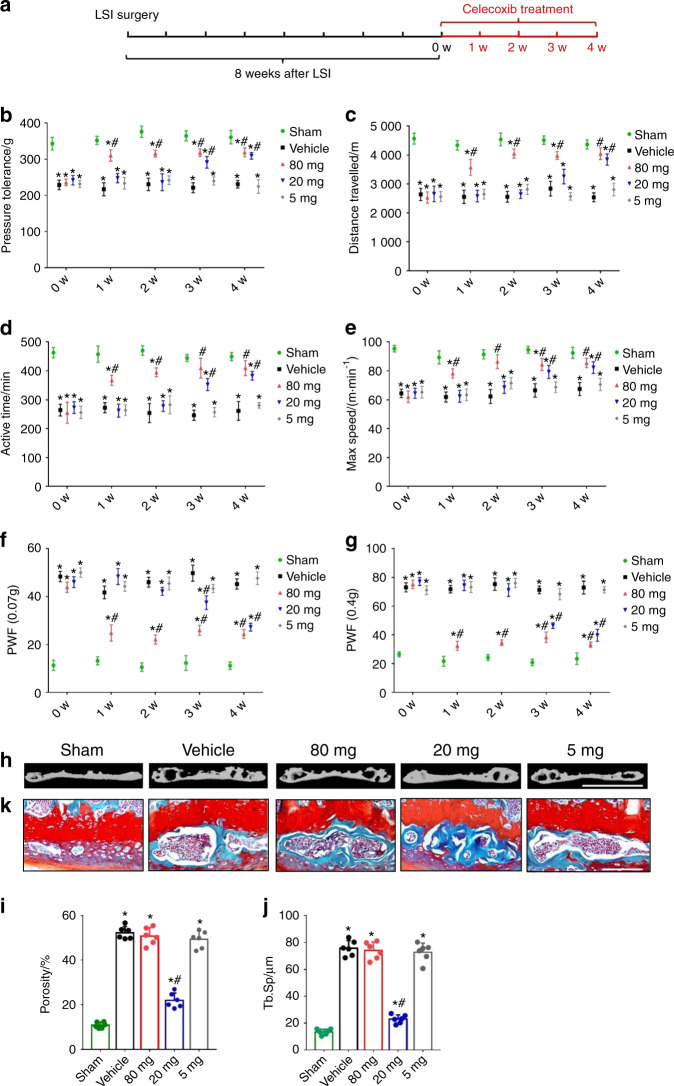
Low-dose celecoxib reduced spinal pain by modifying endplate porosity in the mice with LSI. **a** Different dosages of celecoxib (very low dose, 5 mg·kg^−1^ per day; low dose, 20 mg·kg^−1^ per day; and high dose, 80 mg·kg^−1^ per day) were administered intragastrically in mouse models of lumbar spine instability for 1–4 weeks. **b** Vocalization thresholds of the mice with LSI surgery at 0–4 weeks after celecoxib or vehicle treatment. Spontaneous activity results, including distance traveled (**c**), active time per 24 h (**d**), and maximum speed (**e**). **f**, **g** PWF by the von Frey test (0.07 g and 0.4 g). **h** Representative μCT images of the L4-L5 caudal endplates at 4 weeks after celecoxib or vehicle treatment. Scale bars, 1 mm. Quantitative analysis of the total porosity (**i**) and trabecular separation (**j**) of the L4-L5 caudal endplates based on the μCT images. *P < 0.05 compared with the sham group and ^#^*P* < 0.05 com*p*ared with the vehicle group at the corresponding time points. *n* = 6 per group. **k** Representative safranin O and fast green staining images of the L4-L5 caudal endplate sections at 4 weeks after celecoxib or vehicle treatment. Scale bars, 50 μm

To examine whether low-dose celecoxib treatment reduces vertebral endplate porosity to inhibit pain, we administered 3 dosages of celecoxib (very low dose, 5 mg·kg^−1^ per day; low dose, 20 mg·kg^−1^ per day; and high dose, 80 mg·kg^−1^ per day) intragastrically to mice with LSI for 1 to 4 weeks (Fig. 1a). High-dose celecoxib significantly increased pressure tolerance at 1, 2, 3, and 4 weeks after treatment compared with the vehicle, whereas very low-dose celecoxib had no effect (Fig. 1b). The sham group had no changes in behavior with either high-dose or low-dose celecoxib treatment (Supplementary Fig. 1A–F). Importantly, low-dose celecoxib did not reduce pain hypersensitivity at 1 and 2 weeks after treatment but significantly relieved pain at 3 and 4 weeks (Fig. 1b), suggesting that it may relieve pain through a different mechanism than high-dose celecoxib.

We further examined the drug effects on animals’ voluntary and spontaneous activity, including distance traveled, active time per 24 h, and maximum speed of movement. All three measures increased significantly at 1 to 4 weeks after treatment with high-dose but not very low-dose celecoxib (Fig. 1c–e). Mice that received low-dose celecoxib showed significant increases in these measures only at weeks 3 and 4 (Fig. 1c–e).

Finally, we evaluated the mechanical hypersensitivity of the hind paw by the von Frey test. Similar to the pressure tolerance results, the paw withdrawal frequency (PWF) was significantly reduced by high-dose celecoxib treatment at 1–4 weeks. Low-dose celecoxib gradually decreased mechanical hyperalgesia at 3 and 4 weeks after treatment. Mice receiving very low-dose celecoxib showed no significant changes (Fig. 1f, g).

To understand the delayed pain inhibition of low-dose celecoxib, we examined the caudal endplates of L4-L5 after 4 weeks of treatment using 3-dimensional μCT and histological staining. Celecoxib at a low dose significantly reduced endplate porosity compared to high-dose, very low-dose, and vehicle treatment, as shown by μCT imaging (Fig. 1h–j). Moreover, safranin O and fast green staining of L4-L5 caudal endplates showed smaller endplate cavities in the low-dose group than in the high-dose, very low-dose, and vehicle groups (Fig. 1k). Different dosages of celecoxib had no effect on the bone mass of the lumbar vertebral body in the mice with LSI (Supplementary Fig. 2A–E). Above all, these findings showed that low-dose celecoxib treatment reduces endplate porosity and induces latent inhibition of LBP.

### Celecoxib at a low dose that maintains physiological PGE2 concentrations induced new bone formation in porous endplates

**Fig. 2:**
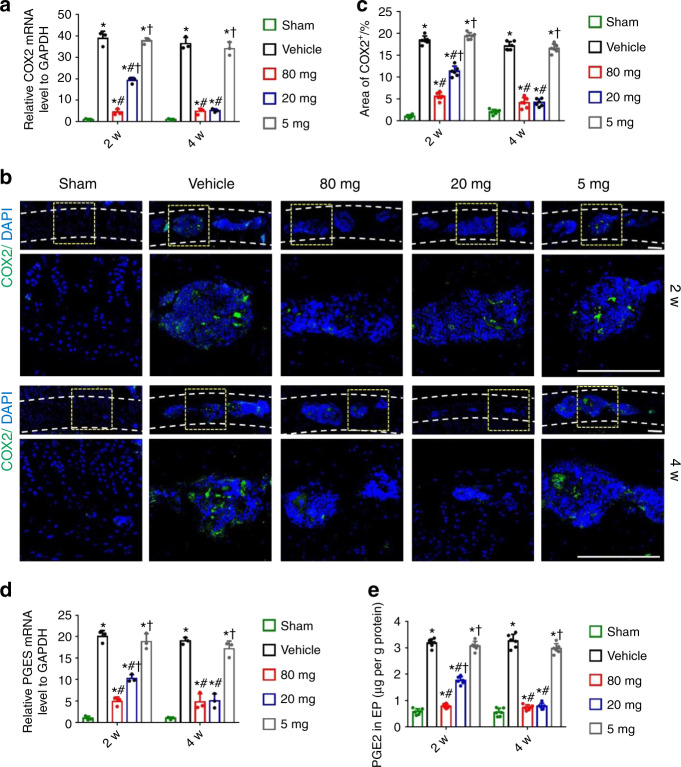
Low-dose celecoxib maintained PGE2 concentrations within the physiological range. **a** Quantitative analysis of COX-2 mRNA expression in lumbar endplates at 2 and 4 weeks after celecoxib or vehicle treatment by qRT-PCR. **b** Representative images of immunostaining of COX-2 (green) and DAPI (blue) in the L4-L5 caudal endplates at 2 and 4 weeks after celecoxib or vehicle treatment. Scale bars, 50 μm. **c** Quantitative analysis of the percentage of COX-2^+^ area in lumbar endplates. **d** Quantitative analysis of PGES mRNA expression in lumbar endplates at 2 and 4 weeks after celecoxib or vehicle treatment by qRT-PCR. **e** ELISAs of the PGE2 concentration in lumbar endplates at 2 and 4 weeks after celecoxib or vehicle treatment. *P < 0.05 compared with the sham group, ^#^*P* < 0.05 com*p*ared with the vehicle group, and ^†^*P* < 0.05 compared with the high-dose group at the corresponding time points. *n* = 3 per group (**a**, **d**); *n* = 6 per group (**c**, **e**)

Our previous study showed that PGE2 produced by COX-2 regulates bone homeostasis by attenuating sympathetic tone^27^. Here, we postulate that new bone formation in porous endplates may be promoted by a physiological concentration of PGE2 as a potential mechanism to reduce sensory innervation and spinal pain. Given that celecoxib is a selective COX-2 inhibitor, we further examined COX-2 expression and PGE2 concentration in the endplates of L4-L5 cells after 2 or 4 weeks of celecoxib treatment. Quantitative real-time polymerase chain reaction (qRT-PCR) and immunostaining showed a decrease in COX-2 at 2 and 4 weeks after high-dose celecoxib treatment relative to that in the control groups (Fig. 2a–c). COX-2 expression partially decreased at 2 weeks following low-dose celecoxib and was further reduced to a similar level as that achieved by high-dose treatment at 4 weeks (Fig. 2a–c). Prostaglandin E synthase (PGES) mRNA levels and PGE2 concentrations were significantly decreased after 2 weeks of high-dose celecoxib treatment, as shown by qRT-PCR and ELISAs, relative to low-dose, very low-dose and vehicle treatment. Importantly, low-dose celecoxib also decreased the PGE2 concentration, which was comparable to that after high-dose celecoxib treatment at 4 weeks (Fig. 2d, e).

The PGE2 concentration in the lumbar vertebral body of the sham group was considered the physiological level for bone homeostasis. We found that the PGE2 concentration in the lumbar vertebral body was not significantly changed with different dosages of celecoxib treatment (Supplementary Fig. 2F), whereas PGE2 concentration in the porous endplate was significantly decreased after 2 weeks of low-dose celecoxib treatment, which was comparable to the PGE2 concentration in the L5 vertebral body (Supplementary Fig. 2F). Thus, the PGE2 concentration in porous endplates was maintained at a physiological level at 2 weeks after low-dose celecoxib treatment.

**Fig. 3:**
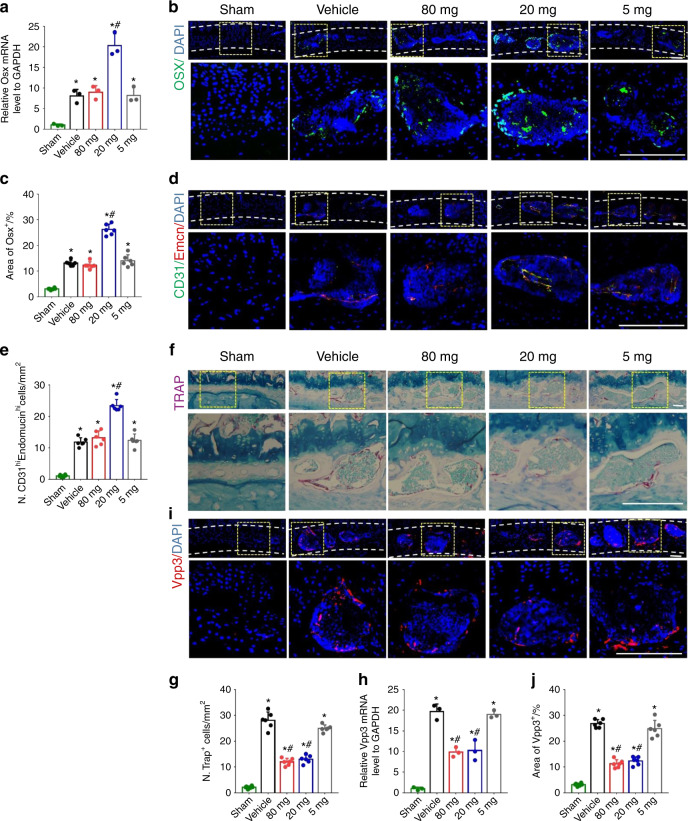
Low-dose celecoxib induced new bone formation in porous endplates. **a** Quantitative analysis of OSX mRNA expression in lumbar endplates at 2 and 4 weeks after celecoxib or vehicle treatment by qRT-PCR. **b** Representative images of immunostaining of osterix (green) and DAPI (blue) in the L4-L5 caudal endplates at 2 weeks after celecoxib or vehicle treatment. **c** Quantitative analysis of the percentage of osterix^+^ area in lumbar endplates. **d** Representative images of immunostaining of CD31 (green), endomucin (red), and DAPI (blue) in the L4-L5 caudal endplates at 2 weeks after celecoxib or vehicle treatment. **e** Quantitative analysis of the number of CD31^hi^endomucin^hi^ cells in lumbar endplates. **f** Representative images of TRAP staining (magenta) in the L4-L5 caudal endplates at 2 weeks after celecoxib or vehicle treatment. **g** Quantitative analysis of the number of TRAP^+^ cells in lumbar endplates. **h** Quantitative analysis of Vpp3 mRNA expression in lumbar endplates at 2 and 4 weeks after celecoxib or vehicle treatment by qRT-PCR. **i** Representative images of immunostaining of Vpp3 (red) and DAPI (blue) in the L4-L5 caudal endplates at 2 weeks after celecoxib or vehicle treatment. **j** Quantitative analysis of the percentage of Vpp3^+^ area in lumbar endplates. Scale bars, 50 μm (**b**, **d**, **f**, **i**). *P < 0.05 compared with the sham group and ^#^*P* < 0.05 com*p*ared with the vehicle group. *n* = 3 per group (**a**, **h**); *n* = 6 per group (**c**, **e**, **g**, **j**)

Since low-dose celecoxib reduced endplate porosity, we then analyzed new bone formation and type H vessel angiogenesis. Osterix expression increased significantly in the low-dose group compared with the high-dose, very low-dose, and vehicle groups, indicating new bone formation only in the low-dose group (Fig. 3a–c). Moreover, immunostaining of CD31^hi^EMCN^hi^ type H vessels showed that type H vessels were significantly more abundant in the low-dose group than in any other group (Fig. 3d, e). Tartrate-resistant acid phosphatase (TRAP) staining showed that the TRAP^+^ cell number in the endplate decreased significantly in the high-dose and low-dose groups after 2 weeks of treatment compared to the very low-dose and vehicle groups. However, there was no significant difference between the high-dose and low-dose groups (Fig. 3f, g), which was further validated with qRT-PCR and immunostaining of Vpp3 for osteoclast function (Fig. 3h–j). These findings indicated that low-dose celecoxib (20 mg·kg^−1^ per day) may promote new bone formation in porous vertebral endplates to reduce porosity.

### Low-dose celecoxib reduced endplate porosity by modulating skeletal interoception

**Fig. 4:**
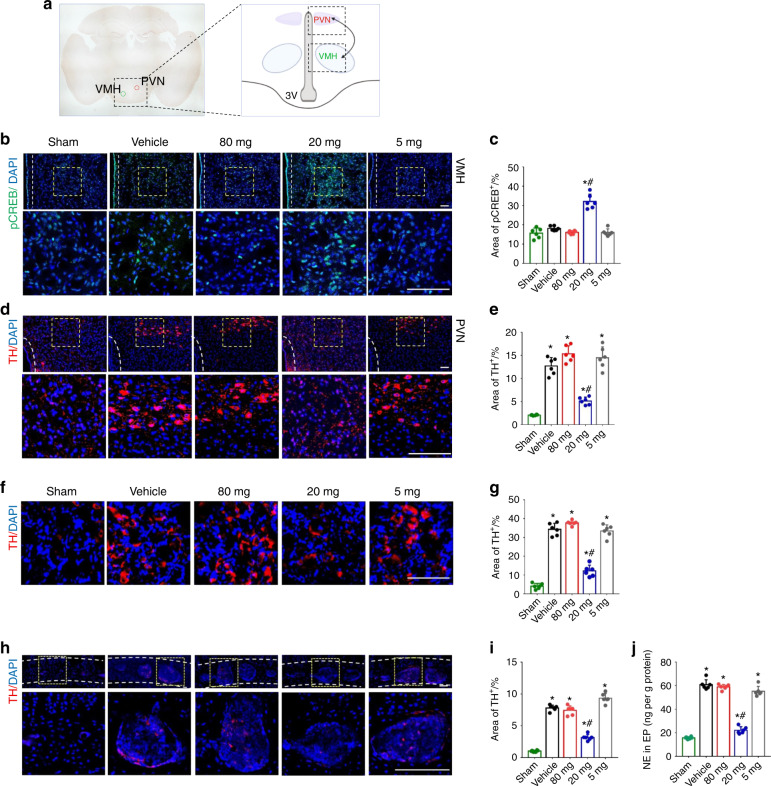
Low-dose celecoxib reduced endplate porosity by decreasing sympathetic activity through hypothalamic CREB signaling. **a** Location of the ventromedial hypothalamic nucleus (VMH) and the paraventricular nucleus (PVN) in the hypothalamus. **b** Representative images of immunostaining of pCREB (green) and DAPI (blue) in the VMH at 2 weeks after celecoxib or vehicle treatment. **c** Quantitative analysis of the percentage of pCREB^+^ area in the VMH. **d** Representative images of immunostaining of TH (red) and DAPI (blue) in the hypothalamic PVN at 2 weeks after celecoxib or vehicle treatment. **e** Quantitative analysis of the percentage of TH^+^ area in the PVN. **f** Representative images of immunostaining of TH (red) and DAPI (blue) in the cervicothoracic ganglion at 2 weeks after celecoxib or vehicle treatment. **g** Quantitative analysis of the percentage of TH^+^ area in the cervicothoracic ganglion. **h** Representative images of immunostaining of TH (red) and DAPI (blue) in the endplates at 2 weeks after celecoxib or vehicle treatment. **i** Quantitative analysis of the percentage of TH^+^ area in endplates. **J** ELISAs of norepinephrine concentration in lumbar endplates at 2 weeks after celecoxib or vehicle treatment. Scale bars, 50 μm (**b, d, f, h**). *P < 0.05 compared with the sham group and ^#^*P* < 0.05 com*p*ared with the vehicle group at the corresponding time points. *n* = 6 per group (**c, e, g, i, j**)

PGE2, which can be produced by osteoblastic cells, activates EP4 receptors on sensory nerves to induce bone formation, which involves decreasing sympathetic tone via hypothalamic CREB signaling^27^. Our immunostaining of hypothalamus sections showed that administration of low-dose (20 mg·kg^−1^ per day) celecoxib significantly increased the phosphorylated CREB (pCREB) level in the ventromedial hypothalamus (VMH) compared with that in any other group (Fig. 4a–c). To evaluate the role of the sympathetic system, we performed immunostaining for tyrosine hydroxylase (TH) for norepinephrine synthesis in the paraventricular nucleus (PVN) of the hypothalamus, cervicothoracic ganglion, and cavities of endplates. TH expression was significantly decreased in the low-dose group compared to the high-dose, very low-dose, and vehicle groups (Fig. 4d–i). Furthermore, we measured the norepinephrine concentration in the endplates, which was significantly decreased in the low-dose group versus any other group (Fig. 4j). These findings suggest that low-dose celecoxib may help to maintain a physiological level of PGE2 to induce bone formation in porous endplates by modulating skeletal interaction.

### Knockout of the EP4 receptor in the sensory nerve abolished the effect of celecoxib on reducing endplate porosity

**Fig. 5:**
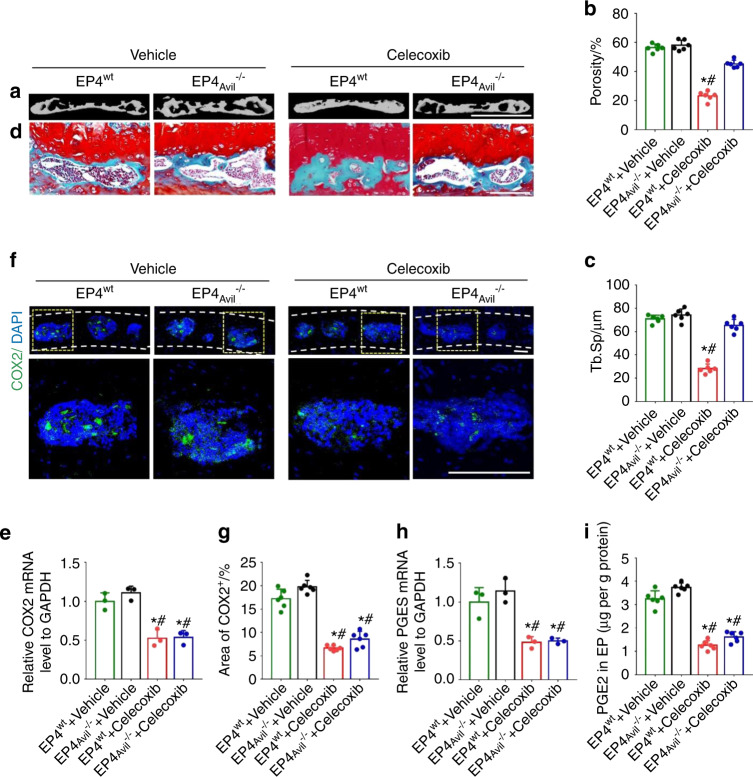
Knockout of the EP4 receptor in the sensory nerves eliminated the effect of low-dose celecoxib on the reduction of endplate porosity. **a** Representative μCT images of the L4-L5 caudal endplates in the EP4^wt^ or EP4_Avil_^−/−^ mice at 4 weeks after celecoxib or vehicle treatment. Scale bars, 1 mm. Quantitative analysis of the total porosity (**b**) and trabecular separation (**c**) of the L4-L5 caudal endplates based on the μCT images. **d** Representative images of safranin O and fast green staining of the L4-L5 caudal endplates in the EP4^wt^ or EP4_Avil_^−/−^ mice at 4 weeks after celecoxib or vehicle treatment. **e** Quantitative analysis of COX-2 mRNA expression in lumbar endplates in the EP4^wt^ or EP4_Avil_^−/−^ mice at 2 weeks after celecoxib or vehicle treatment by qRT-PCR. **f** Representative images of immunostaining of COX-2 (green) and DAPI (blue) in the L4-L5 caudal endplates in the EP4^wt^ or EP4_Avil_^−/−^ mice at 2 weeks after celecoxib or vehicle treatment. **g** Quantitative analysis of the percentage of COX-2^+^ area in lumbar endplates. **h** Quantitative analysis of PGES mRNA expression in lumbar endplates in the EP4^wt^ or EP4_Avil_^−/−^ mice at 2 weeks after celecoxib or vehicle treatment. **i** ELISAs of PGE2 concentration in lumbar endplates in the EP4^wt^ or EP4_Avil_^−/−^ mice at 2 weeks after celecoxib or vehicle treatment. Scale bars, 50 μm (**d, f**). *P < 0.05 compared with the sham group and ^#^*P* < 0.05 compared with the vehicle group at the corresponding time points. *n* = 3 per group (**e**, **h**); *n* = 6 per group (**b, c, g, i**)

**Fig. 6:**
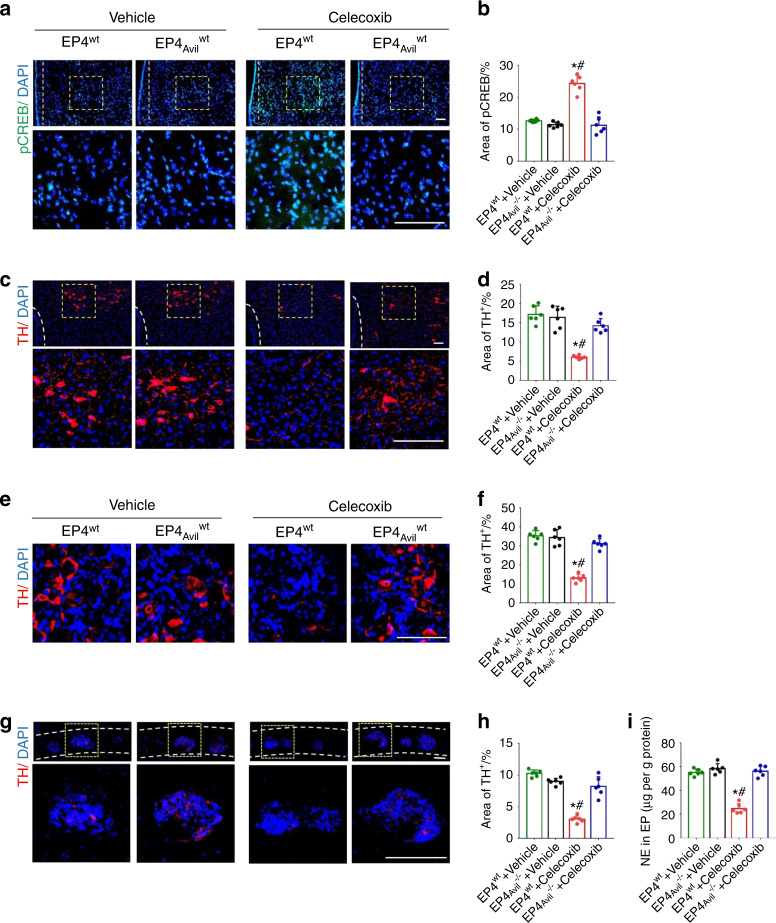
Knockout of the EP4 receptor in the sensory nerves influenced the effect of low-dose celecoxib on pCREB expression in the VMH and TH expression in the PVN, cervicothoracic ganglion and endplate. **a** Representative images of immunostaining of pCREB (green) and DAPI (blue) in hypothalamic VMH in the EP4^wt^ or EP4_Avil_^−/−^ mice at 2 weeks after celecoxib or vehicle treatment. **b** Quantitative analysis of the pCREB^+^ area in the VMH. **c** Representative images of immunostaining of TH (red) and DAPI (blue) in the hypothalamic PVN in the EP4^wt^ or EP4_Avil_^−/−^ mice at 2 weeks after celecoxib or vehicle treatment. **d** Quantitative analysis of the TH^+^ area in the PVN. **e** Representative images of immunostaining of TH (red) and DAPI (blue) in the cervicothoracic ganglion of the EP4^wt^ or EP4_Avil_^−/−^ mice at 2 weeks after celecoxib or vehicle treatment. **f** Quantitative analysis of the TH^+^ area in the cervicothoracic ganglion. **g** Representative images of immunostaining of TH (red) and DAPI (blue) in the endplates of the EP4^wt^ or EP4_Avil_^−/−^ mice at 2 weeks after celecoxib or vehicle treatment. **h** Quantitative analysis of the TH^+^ area in endplates. **i** ELISAs of norepinephrine concentration in lumbar endplates in the EP4^wt^ or EP4_Avil_^−/−^ mice at 2 weeks after celecoxib or vehicle treatment. Scale bars, 50 μm (**a, c, e, g**). *P < 0.05 compared with the sham group and ^#^*P* < 0.05 compared with the vehicle group at the corresponding time points. *n* = 6 per group (**b**, **d**, **f**, **h**, **i**)

To determine whether bone formation in porous vertebral endplates is mediated by PGE2-EP4 signaling in sensory nerves, we crossed Advillin-Cre mice with EP4^flox/flox^ mice to generate Advillin-Cre; EP4^flox/flox^ mice, in which EP4 receptor expression was specifically deleted in primary sensory neurons. Herein, these mice are referred to as EP4_Avil_^−/−^. In the EP4^wt^ mice with LSI, low-dose celecoxib (20 mg·kg^−1^ per day) significantly reduced endplate porosity relative to that of the vehicle group, as shown in μCT analysis. However, this effect was diminished in the EP4_Avil_^−/−^ mice (Fig. 5a–c). Safranin O and fast green staining of endplate sections also showed that low-dose celecoxib significantly decreased endplate porosity in the EP4^wt^ mice with LSI but not in the EP4_Avil_^−/−^ mice (Fig. 5d). COX-2 expression (Fig. 5e–g), PGES expression (Fig. 5h) and PGE2 concentration (Fig. 5i) in the porous endplates decreased similarly in the EP4^wt^ and EP4_Avil_^−/−^ mice administered low-dose celecoxib. Collectively, these findings suggest that PGE2 signaling through EP4 receptors in sensory nerves may mediate the inhibition of endplate porosity by low-dose celecoxib. Immunostaining results showed that pCREB in the hypothalamus was increased by low-dose celecoxib treatment in the EP4^wt^ mice compared to that of the vehicle group. In contrast, the hypothalamic pCREB level in the EP4_Avil_^−/−^ mice was comparable to that of the vehicle group (Fig. 6a, b). As expected, low-dose celecoxib decreased TH expression in the PVN, cervicothoracic ganglion, and cavities of the endplates in the EP4^wt^ mice but not the EP4_Avil_^−/−^ mice (Fig. 6c–h). Finally, we measured the norepinephrine concentration in the endplates because it indicates sympathetic functional activity. The norepinephrine concentration was also reduced significantly by low-dose celecoxib in the EP4^wt^ mice but not in the EP4_Avil_^−/−^ mice compared to vehicle (Fig. 6i). Thus, low-dose celecoxib may maintain a physiological concentration of PGE2 in porous vertebral endplates to activate the skeletal interoception pathway, thereby reducing endplate porosity.

### Reduction of endplate porosity and relief of spinal pain remained after discontinuing low-dose celecoxib treatment

**Fig. 7:**
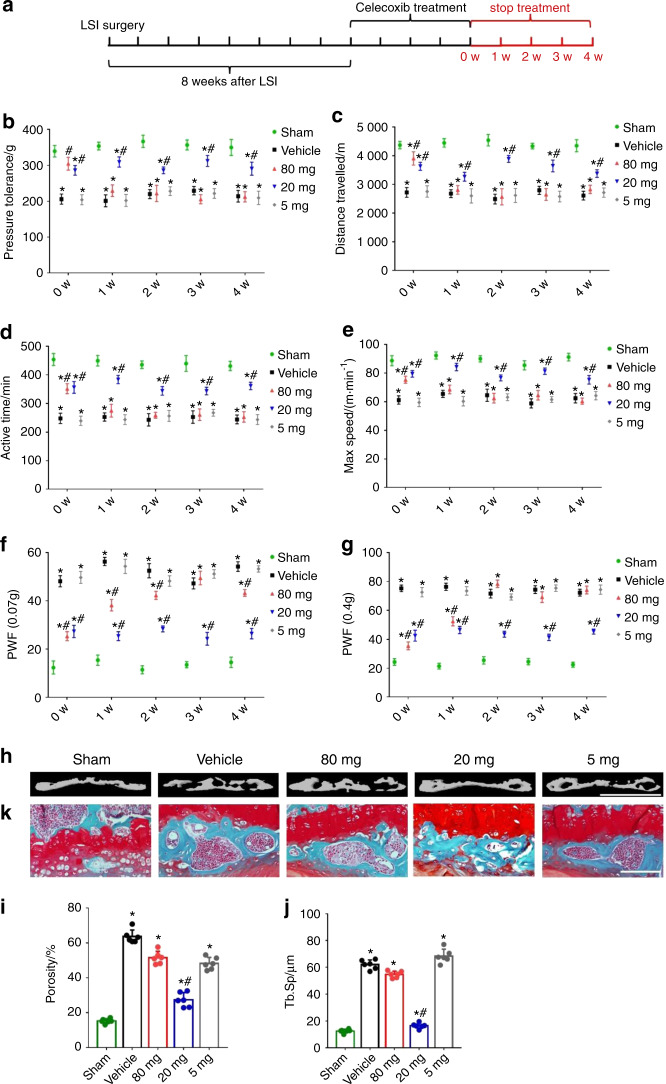
Spinal pain hypersensitivity remained relieved after discontinuation of low-dose celecoxib treatment. **a** Spinal pain and endplate porosity were measured at 4 weeks after discontinuation of celecoxib or vehicle treatment. **b** Vocalization thresholds of the mice with LSI surgery at 4 weeks after the discontinuation of celecoxib or vehicle treatment. Spontaneous activity results, including distance traveled (**c**), active time per 24 h (**d**), and maximum speed (**e**). **f**, **g** The PWF in the von Frey test (0.07 g and 0.4 g). **h** Representative μCT images of the L4-L5 caudal endplates at 4 weeks after the discontinuation of celecoxib or vehicle treatment. Scale bars, 1 mm. Quantitative analysis of the total porosity (**i**) and trabecular separation (**j**) of the L4-L5 caudal endplates based on the μCT images. *P < 0.05 compared with the sham group and ^#^*P* < 0.05 com*p*ared with the vehicle group at the corresponding time points. *n* = 6 per group. **k** Representative safranin O and fast green staining images of the L4-L5 caudal endplate sections at 4 weeks after the discontinuation of celecoxib or vehicle treatment. Scale bars, 50 μm

We further investigated whether the reduction in endplate porosity by low-dose celecoxib is associated with an attenuation of LBP. The mice with LSI received different dosages of celecoxib for 4 weeks. Spinal pain and endplate porosity were measured at 1, 2, 3, or 4 weeks after discontinuation of the treatment (Fig. 7a). In pressure tolerance and spontaneous activity tests, the beneficial effects of high-dose celecoxib diminished quickly beginning at 1 week after discontinuation of the treatment. However, the increase in pressure tolerance and spontaneous activity persisted in mice after discontinuation of low-dose celecoxib treatment (Fig. 7b–e). Similarly, the inhibitory effect of low-dose celecoxib on hind paw mechanical hypersensitivity, as indicated by decreased PWFs to 0.07 g or 0.4 g stimulation, was also sustained for 4 weeks after discontinuation of treatment. In contrast, the inhibition of mechanical hypersensitivity by high-dose celecoxib rapidly diminished after discontinuation of drug treatment (Fig. 7f, g). These results indicated that low-dose celecoxib may perpetually reduce spinal pain and referred mechanical hypersensitivity. High-dose celecoxib induced temporary pain inhibition by decreasing COX-2 activity and PGE2 concentration during the period of drug treatment.

To validate these results, we analyzed the porosity of the L4-L5 caudal endplates 4 weeks after discontinuing drug treatment by conducting μCT and immunohistology studies. Endplate porosity was high in the high-dose and very low-dose groups at this time point. In contrast, endplate porosity in the low-dose group was significantly lower than that in the high-dose, very low-dose and vehicle groups (Fig. 7h–j). Moreover, safranin O and fast green staining of endplate sections confirmed these findings from μCT imaging (Fig. 7k). These results suggest that endplate porosity was reduced for a long time period after treatment with low-dose celecoxib, which may contribute to its prolonged pain inhibition.

### Low-dose celecoxib reduced sensory innervation in porous endplates and TRPV1 expression in DRG neurons

**Fig. 8:**
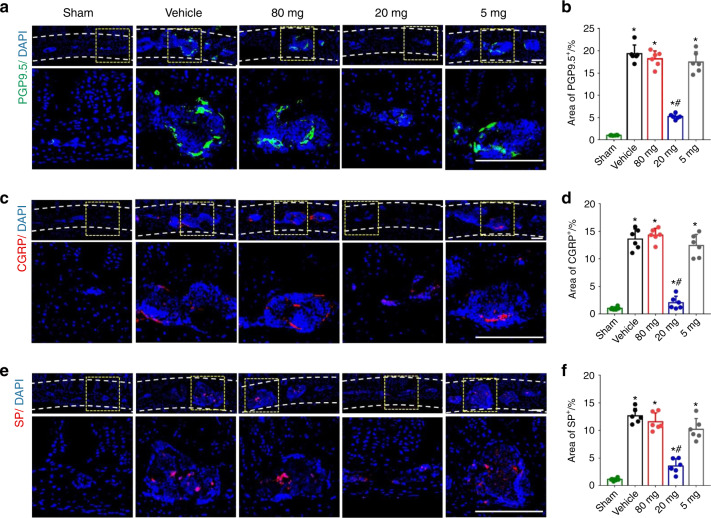
Sensory innervation in the porous endplates was reduced by low-dose celecoxib. **a** Representative images of immunostaining of PGP9.5 (green) and DAPI (blue) of nerve fibers in the lumbar endplates at 4 weeks after the discontinuation of celecoxib or vehicle treatment. **b** Quantitative analysis of the percentage of PGP9.5^+^ area in lumbar endplates. **c** Representative images of immunostaining of CGRP (red) and DAPI (blue) of nerve fibers in the lumbar endplates at 4 weeks after the discontinuation of celecoxib or vehicle treatment. **d** Quantitative analysis of the percentage of CGRP^+^ area in lumbar endplates. **e** Representative images of immunostaining of SP (red) and DAPI (blue) in the lumbar endplates at 4 weeks after the discontinuation of celecoxib or vehicle treatment. **f** Quantitative analysis of the percentage of SP^+^ area in lumbar endplates. Scale bars, 50 μm (**a, c, e**) *P < 0.05 compared with the sham group and ^#^*P* < 0.05 com*p*ared with the vehicle group at the corresponding time points. *n* = 6 per group (**b, d, f**)

**Fig. 9:**
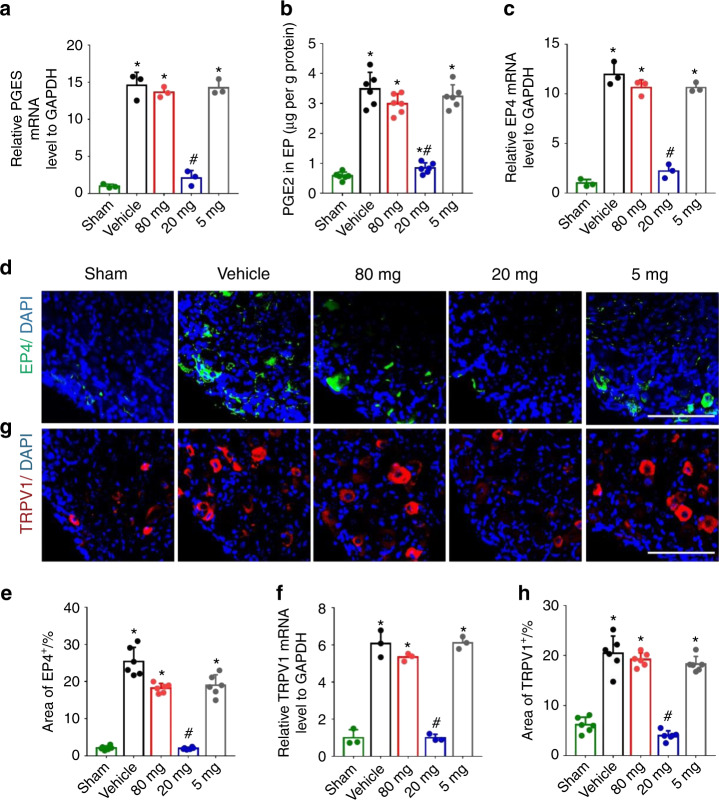
EP4 and TRPV1 expression in dorsal root ganglion (DRG) neurons was reduced by low-dose celecoxib. **a** Quantitative analysis of PGES mRNA expression in lumbar endplates at 4 weeks after the discontinuation of celecoxib or vehicle treatment. **b** ELISAs of PGE2 concentration in lumbar endplates at 4 weeks after the discontinuation of celecoxib or vehicle treatment. **c** Quantitative analysis of EP4 mRNA expression in the L2 DRGs at 4 weeks after the discontinuation of celecoxib or vehicle treatment. **d** Representative images of immunostaining of EP4 (green) and DAPI (blue) in the L2 DRG at 4 weeks after the discontinuation of celecoxib or vehicle treatment. **e** Quantitative analysis of the percentage of EP4^+^ area in L2 DRGs. **f** Quantitative analysis of TRPV1 mRNA expression in the L2 DRGs at 4 weeks after the discontinuation of celecoxib or vehicle treatment. **g** Representative images of immunostaining of TRPV1 (red) and DAPI (blue) in the L2 DRG at 4 weeks after the discontinuation of celecoxib or vehicle treatment. **h** Quantitative analysis of the percentage of TRPV1^+^ area in L2 DRGs. Scale bars, 50 μm (**d, g**). *P < 0.05 compared with the sham group and ^#^*P* < 0.05 compared with the vehicle group at the corresponding time points. *n* = 3 per group (**a, c, f**); *n* = 6 per group (**b, e, h**)

To further investigate the neuronal mechanism by which reduced endplate porosity is associated with a decrease in spinal pain, we examined the effect of low-dose (20 mg·kg^−1^ per day) celecoxib on sensory innervations in porous vertebral endplates. Immunofluorescence staining showed a high level of PGP9.5^+^ and CGRP^+^ sensory nerve innervation in the porous endplates of the mice with LSI with high-dose, very low-dose or vehicle treatment. Importantly, PGP9.5^+^ and CGRP^+^ innervation was significantly decreased in the low-dose group compared to the other groups (Fig. 8a–d). Furthermore, immunostaining of substance P, which is expressed in nociceptive neurons (Fig. 8e, f), PGES expression and PGE2 concentration in the porous endplates were significantly decreased in the low-dose celecoxib group (Fig. 9a, b). TRPV1 is associated with inflammatory pain and heat pain and is implicated in downstream PGE2/EP4 signaling^26,49^. qRT-PCR and immunostaining showed that low-dose celecoxib reduced the expression of TRPV1 and EP4 in L2 DRGs. However, their expression levels were significantly increased in the DRGs from the mice with LSI treated with high-dose celecoxib, very low-dose celecoxib, or vehicle (Fig. 9c–h). These results revealed that low-dose celecoxib may reduce painful sensory innervation and the expression of EP4 and TRPV1 in the vertebral endplates of the mice with LSI.

## Discussion

**Fig. 10:**
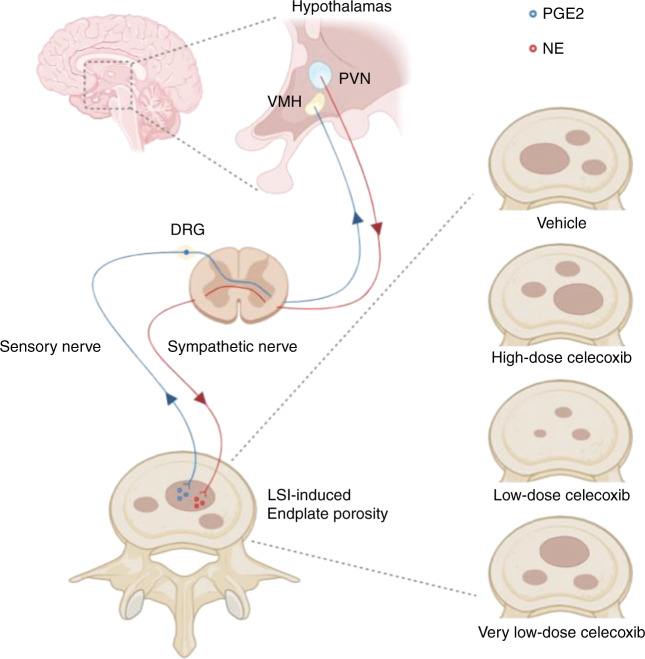
Diagram of PGE2/EP4-mediated skeletal interoception-induced modulation of endplate porosity and LBP. Activation of EP4 in sensory nerves by PGE2 induces osteoblastic bone formation through the hypothalamus to inhibit sympathetic activity in a manner similar to PGE2/EP4-mediated skeletal interoception.^27,29^ Endplates undergo sclerosis and become porous by osteoclast resorption during aging or spine degeneration.^16,20^ Low-dose celecoxib promotes bone formation in porous vertebral endplates by maintaining a physiological PGE2 concentration to activate skeletal interoception. However, very low PGE2 levels in porous endplates with high-dose celecoxib could not activate skeletal interoception, and high PGE2 levels with vehicle or very low-dose celecoxib treatment induced pain

The current study is a focused investigation of the effect of celecoxib on porous endplates entirely based on our recent studies.^16,20,27,29^ We have shown that activation of EP4 in sensory nerves by PGE2 induces osteoblastic bone formation through the hypothalamus to inhibit sympathetic activity in a manner similar to PGE2/EP4-mediated skeletal interoception.^27,29^ Endplates undergo sclerosis and become porous by osteoclast resorption during aging or spine degeneration^16,20^ (Fig. 10).

Spinal pain is a major clinical problem; however, its origins and underlying mechanisms remain unclear. There are many factors, including IVD, endplate and facet joints, that are involved in LBP. Endplates undergo ossification and become highly porous during IVD degeneration^16,17^ with more nerve innervation in degenerative endplates than in healthy endplates.^19^ Recent clinical studies have revealed that Modic changes in endplates and vertebral bone marrow lesions on MRI or CT are associated with LBP, which provides potential diagnostic and treatment for LBP related to the porosity of endplates.^10,50,51^ Elevated PGE2 concentrations and increased sensory nerve innervation exist in porous and sclerotic vertebral endplates, and PGE2 activates EP4 receptors in the sprouting nerves to cause spinal pain.^20^ In this study, celecoxib at a low dose (20 mg·kg^−1^ per day), which is equivalent to one-quarter of the maximum dosage used in the clinic, induced prolonged inhibition of LBP and reduced endplate porosity in the mice with LSI. Although high-dose celecoxib (80 mg·kg^−1^ per day) also inhibited LBP, the effect diminished quickly after discontinuing the treatment and did not modify the disease (Fig. 10).

We have established PGE2/EP4-mediated skeletal interoception in the regulation of bone homeostasis.^27^ Current findings in the mice with LSI suggest that low-dose celecoxib may improve bone formation in porous vertebral endplates by maintaining a physiological PGE2 concentration. This beneficial effect may involve activation of skeletal interoception and central mechanisms, as indicated by increased pCREB levels in the VMH and a decrease in TH levels in the PVN. However, celecoxib at a high dose resulted in a very low level of PGE2, which could not activate skeletal interoception or induce bone formation in porous vertebral endplates. COX-2 inhibitors, including celecoxib, have been used widely to treat skeletal pain conditions.^52^ However, COX-2 inhibitors may cause various adverse effects at clinical dosages and thus limit their use. Here, we showed that celecoxib, at one-quarter of the maximum clinical dose, not only induced prolonged pain inhibition but also modified the underlying pathology of LBP in the mice with LSI, as indicated by a decrease in endplate porosity after treatment. In contrast, neither the higher dose nor the lower dosage of celecoxib exerted this effect.

Our data suggest that low-dose celecoxib may exert these effects by restoring or activating skeletal interoception in the mice with LSI. Normal skeletal interoception requires maintaining a proper local PGE2 concentration as a fundamental mechanism for the central nervous system to control bone homeostasis. Celecoxib inhibits COX-2 expression during the synthesis of PGE2, which regulates skeletal interoception and may modify LBP. Our findings also suggest that PGE2 at physiological concentrations could activate skeletal interoception, regulating bone homeostasis. In contrast, very low PGE2 levels in porous endplates after high dose-celecoxib treatment could not activate skeletal interoception, and high PGE2 levels after very low dose-celecoxib or vehicle treatment induced pain and did not activate skeletal interoception. Therefore, high-dose or very low-dose celecoxib or vehicle treatment could not improve bone formation in porous endplates. Most importantly, our findings revealed that low-dose COX-2 inhibitors may have the potential to modify the underlying etiology of skeletal disorders to relieve pain through skeletal interoception.

Sensory inputs from the visceral tissues are carried to the central nervous system by spinal and cranial afferents.^28^ Oury et al. reported that CREB expression in VMH neurons favors bone mass accrual by inhibiting the activity of sympathetic neurons.^53^ Takeda et al. reported that the sympathetic nervous system is important to the regulation of bone formation.^54^ Our finding that low-dose celecoxib increased pCREB levels in the VMH and decreased TH expression in the PVN and cervicothoracic ganglion suggests the involvement of skeletal interoception in skeletal diseases and LBP. Indeed, selective knockout of the EP4 receptor in the sensory nerve blocked the changes in pCREB and TH expression in the hypothalamus and the decrease in endplate porosity by low-dose celecoxib treatment. In addition, the decrease in endplate porosity from low-dose celecoxib was accompanied by decreases in sensory nerve innervation and SP secretion after discontinuation of treatment. SP is widely expressed throughout the animal kingdom^55^ and can transmit nociceptive signals via primary sensory fibers to the spinal cord.^56^ PGE2 has been shown to increase the surface trafficking of EP4 and TRPV1 in DRG neurons.^49^ In a rat model of restraint stress, high-level PGE2 generated in damaged tissues increased EP4 and TRPV1 expression in DRG neurons, and EP4 antagonists relieved both inflammatory and neuropathic pain.^26^ We found that both EP4 and TRPV1 expression was significantly decreased with low-dose celecoxib, suggesting that physiological levels of PGE2 promote bone formation in porous endplates to modify the disease through PGE2/EP4 skeletal interoception.

## Materials and methods

### Mice and in vivo treatment

C57BL/6 J (wild-type [WT]) male mice were purchased from Charles River Laboratories (Wilmington, MA). Two-month-old mice were anesthetized by ketamine (100 mg·kg^−1^, intraperitoneally) and xylazine (10 mg·kg^−1^, intraperitoneally). The L3-L5 spinous processes and supraspinous and interspinous ligaments were resected to create the LSI model. The mice in the sham group underwent detachment of the posterior paravertebral muscles from L3-L5.^57^ At 8 weeks after LSI surgery, 4 groups of mice received vehicle, 80 mg·kg^−1^ per day celecoxib (Sigma-Aldrich, PZ0008), 20 mg·kg^−1^ per day celecoxib, or 5 mg·kg^−1^ per day celecoxib by oral administration. The 80 mg·kg^−1^ per day dosage in mice is equal to the maximum dosage of 400 mg·d^−1^ in humans for a 60-kg person.^58^ We euthanized the mice by using an overdose of isoflurane at 2 or 4 weeks after celecoxib or vehicle treatment (10–12 mice per group) and at 4 weeks after discontinuation of celecoxib or vehicle treatment (10–12 mice per group).

The Avil-Cre mouse strain was obtained from Dr. Xinzhong Dong (The Johns Hopkins School of Medicine, Baltimore, MD). The EP4^flox/flox^ mouse strain was provided by Dr. Brian L. Kelsall (National Institutes of Health, Bethesda, MD). We crossed heterozygous male Avil-Cre mice with female EP4^flox/flox^ mice. The offspring were then intercrossed to generate the following genotypes: Avil-Cre; EP4^flox/flox^, Avil-Cre, EP4^flox/flox^ and WT. We extracted genomic DNA from the tails of mice and determined the genotypes by using polymerase chain reaction (PCR) with the following primers:

Avil-Cre: forward: 5ʹ-CCCTGTTCACTGTGAGTAGG-3ʹ,

reverse: 5ʹ-GCGATCCCTGAACATGTCCATC-3ʹ,

WT: 5ʹ-AGTATCTGGTAGGTGCTTCCAG-3ʹ;

EP4 loxP allele: forward: 5ʹ-TCTGTGAAGCGAGTCCTTAGGCT-3ʹ,

reverse: 5ʹ-CGCACTCTCTCTCTCCCAAGGAA-3ʹ.

LSI surgery was performed in the EP4^wt^ and EP4_Avil_^−/−^mice at 2 months of age. At 8 weeks after LSI surgery, the EP4^wt^ mice and the EP4_Avil_^−/−^ mice received vehicle or 20 mg·kg^−1^ per day celecoxib orally. We euthanized the mice by using an overdose of isoflurane at 2 or 4 weeks after celecoxib or vehicle treatment (10–12 mice per group). All mice were maintained at the animal facility of the Johns Hopkins University School of Medicine.

### Behavioral testing

We performed three kinds of pain-related behavior tests before celecoxib or vehicle treatment, weekly after the treatment and weekly after the discontinuation of treatment.

Vocalization thresholds were recorded by using a force gauge (Bioseb, Pinellas Park, FL), which could reflect pressure hyperalgesia.^59^ We restrained the animal and pressed its skin over the L4-L5 spine with the sensor tip. We then increased the pressure force at 50 g·s^−1^ until the mouse made a vocalization. Tissue damage should be prevented at an upper limit of the pressure force of 500 g.

We used wheel activity (Bioseb) to evaluate the animals’ spontaneous activity. The cage of the device was similar to the animal’s home cage, and the wheel of the device could be spun by the animal in both directions. Animal spontaneous activity was recorded by an analyzer connected to the device. Three indicators, including distance traveled, total active time and maximum speed, were analyzed in our study.

Von Frey tests (0.07 g and 0.4 g) were used to measure the PWF induced by mechanical stimuli (Stoelting, Wood Dale, IL). Mice were restricted to a clear plastic cage placed on a metal mesh. Before the von Frey test, animals were allowed to adapt to the environment for 30 min. We used von Frey filaments to press the mid-plantar surface of the animal’s hind paw through a metal mesh and recorded each withdrawal after the application. A trial consisted of a von Frey test 10 times at 1-s intervals. PWF was calculated as the percentage of withdrawal times in response to 10 applications.

### μCT

Mice were euthanized by overdose isoflurane and then fixed with 10% buffered formalin. We dissected the lumbar spine and fixed it in 10% buffered formalin (4 °C, 24 h). The samples were scanned by high-resolution μCT (voltage, 55 kVp; current, 181 μA; resolution, 9.0 μm per pixel) (Skyscan 1172; Skyscan US, San Jose, CA). Images were reconstructed by NRecon v1.6 (Skyscan US) and analyzed by CTAn v1.9 (Skyscan US). We used images of the L4-L5 vertebral unit and L5 vertebrae (coronal view) to analyze the endplate and trabecular bone, respectively. A total of six consecutive images were used to show 3-dimensional reconstruction of the endplates and vertebrae by CTVol v2.0 (Skyscan US). Two parameters, including total porosity and trabecular separation, were used to analyze the endplates, and four parameters, trabecular BV/TV, Tb.N, Tb.Th, and Tb.Sp, were used for the analysis of L5 vertebrae.

### Histochemistry, immunofluorescence, and histomorphometry

We dissected the lumbar spine and L2 DRG and fixed the samples in 10% buffered formalin (4 °C, 24 h). The spine samples were decalcified by 0.5 mol·L^−1^ ethylenediaminetetraacetic acid (4 °C, 3 weeks) and then embedded in paraffin or 8% gelatin in the presence of 20% sucrose and 2% polyvinylpyrrolidone. The DRG samples were dehydrated by 30% sucrose (4 °C, 48 h) and then embedded in optimal cutting temperature compound (Sakura Finetek, Torrance, CA). We used 4-μm-thick sections of spine samples for safranin O and fast green and TRAP staining. We used 40-μm-thick sections of spine samples for nerve- and blood vessel-related immunostaining. We used 10-μm-thick sections of spine or DRG samples for other immunostaining. The sections were incubated with primary antibodies against COX-2 (1:100, ab15191, Abcam), Vpp3 (1:100, ab200839, Abcam), Osterix (1:100, ab22552, Abcam), Endomucin (1:50, sc-65495, Santa Cruz Biotechnology), CD31 (1:100, ab28364, Abcam), pCREB (1:100, ab32096, Abcam), TH (1:200, ab152, Abcam), CGRP (1:100, ab81887, Abcam), PGP9.5 (1:100, ab10404, Abcam), EP4 (1:50, ab92763, Abcam), and TRPV1 (1:200, ab6166, Abcam) (4 °C, overnight). Then, the sections were incubated with secondary antibodies in the dark (room temperature, 1 h). We used fluorescence microscopy (Olympus BX51, DP71) or confocal microscopy (Zeiss LSM 780) to capture images and ImageJ (National Institutes of Health, Bethesda, MD) software to analyze the results.

### qRT-PCR

We extracted total RNA from the endplate samples by TRIzol reagent (Invitrogen, Carlsbad, CA). The RNA purity was measured by the absorbance at 260/280 nm. For qRT-PCR, 1 μg RNA was reverse transcribed into complementary DNA using the SuperScript First-Strand Synthesis System (Invitrogen). We then performed qRT-PCR by using SYBR Green Master Mix (Qiagen, Hilden, Germany). Relative expression of mRNA was analyzed by the 2^−ΔΔ^ ^CT^ method. The primers used in the qRT-PCR experiments are listed below:

COX-2: forward: 5ʹ- CAGACAACATAAACTGCGCCTT -3ʹ,

reverse: 5ʹ- GATACACCTCTCCACCAATGACC -3ʹ;

PGES: forward: 5ʹ- TTTCTGCTCTGCAGCACACT -3ʹ,

reverse: 5ʹ- GATTGTCTCCATGTCGTTGC -3ʹ;

OSX: forward: 5ʹ- GGAGGTTTCACTCCATTCCA -3ʹ,

reverse: 5ʹ- TAGAAGGAGCAGGGGACAGA -3ʹ;

VPP3: forward: 5ʹ- ATCAATGTGCTCCCATCCCTCT -3ʹ,

reverse: 5ʹ- AATGCGCTTCAGCATCTCTTTC -3ʹ;

EP4: forward: 5ʹ- CGGTTCCGAGACAGCAAA -3ʹ,

reverse: 5ʹ- CGGTTCGATCTAGGAATGG -3ʹ;

TRPV1: forward: 5ʹ- TCTCCACTGGTGTTGAGACG -3ʹ,

reverse: 5ʹ- GGGTCTTTGAACTCGCTGTC -3ʹ;

GAPDH: forward: 5ʹ- AATGTGTCCGTCGTGGATCTGA -3ʹ,

reverse: 5ʹ- AGTGTAGCCCAAGATGCCCTTC -3ʹ.

### ELISA

We used a PGE2 ELISA Kit (KGE004B, R&D Systems) and Norepinephrine ELISA Kit (BA E-5200, Rocky Mountain Diagnostics, Inc.) to test the PGE2 and norepinephrine concentrations in the L3-L5 endplates.

### Statistical analysis

We used SPSS 15.0 (IBM Corp., Armonk, NY) to perform the statistical analysis. Data are presented as the mean ± standard deviations. One-way ANOVA with Bonferroni’s post hoc test was used for comparisons among multiple groups. Two-way ANOVA with repeated measures was used to evaluate the effects of different doses of celecoxib treatment on spinal hypersensitivity and movements at different time points. Two-way ANOVA with Bonferroni’s post hoc test was used to analyze the effects of low-dose celecoxib on the EP4^wt^ and EP4_Avil_^−/−^mice with LSI surgery. Comparisons of PGE2 concentrations between vertebral bodies and endplates at different dosages in the celecoxib treatment group were also analyzed by two-way ANOVA with Bonferroni’s post hoc test. For all experiments, *P* < 0.05 was considered significant.

### Study approval

All experimental protocols were approved by the Animal Care and Use Committee of The Johns Hopkins University, Baltimore, MD.

## Supplementary information

Supplementary Data

Supplementary information
