# Supplementary figures and images for: PGE2/EP4 skeleton interoception activity reduces vertebral endplate porosity and spinal pain with low-dose celecoxib
Source: Bone Res. 2021 Aug 2;9:36. doi: 10.1038/s41413-021-00155-z (PMC8326284; doi:10.1038/s41413-021-00155-z)

Supplementary Figure 1

A

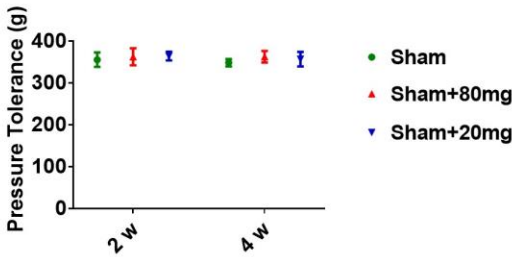

B

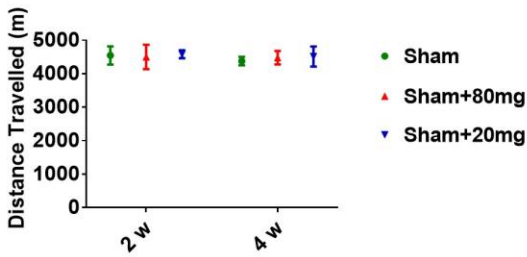

C

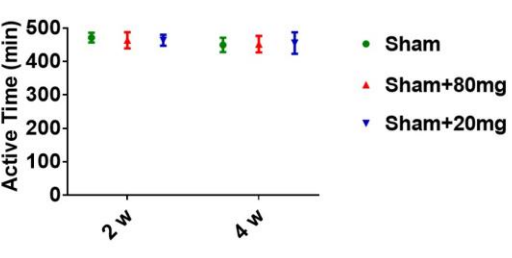

D

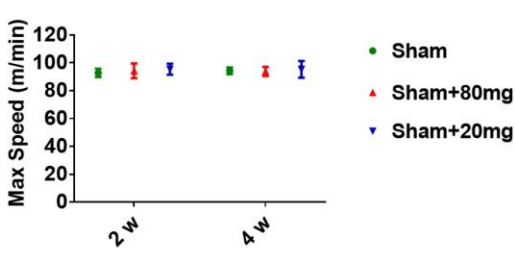

E

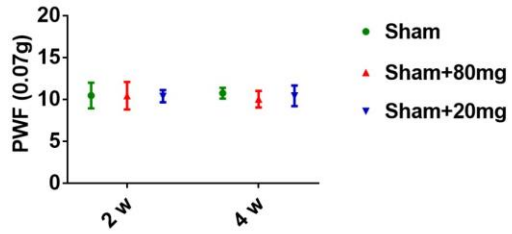

F

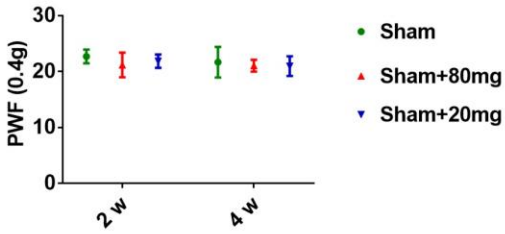

Supplementary Figure 2

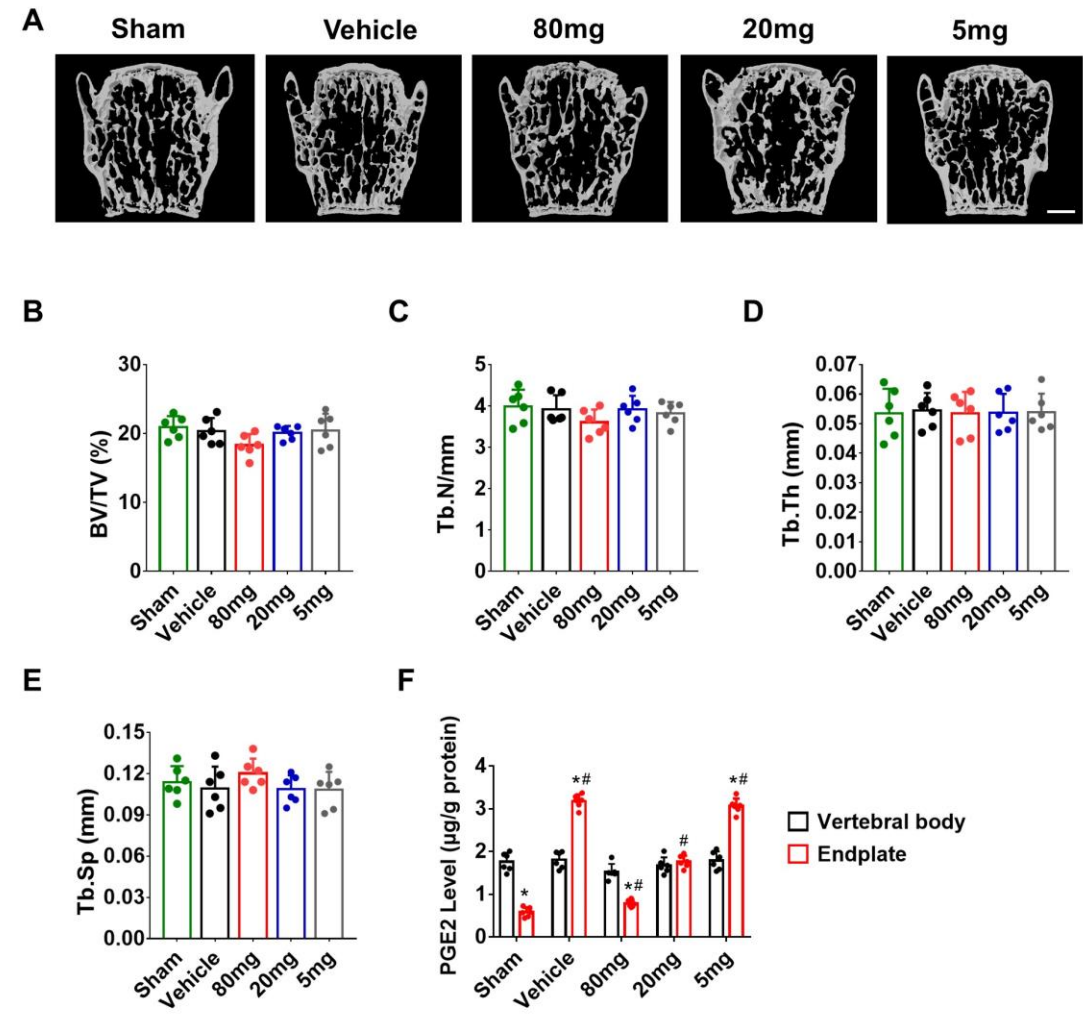

Supplement: Supplementary file 1 — Supplementary Data [file 41413_2021_155_MOESM1_ESM.pdf]
