# Supplementary material for: PGE2/EP4 skeleton interoception activity reduces vertebral endplate porosity and spinal pain with low-dose celecoxib
Source: Bone Res. 2021 Aug 2;9:36. doi: 10.1038/s41413-021-00155-z (PMC8326284; doi:10.1038/s41413-021-00155-z)
Supplement: Supplementary file 2 — Supplementary information [file 41413_2021_155_MOESM2_ESM.docx]

**Supplementary Figure 1. High-dose and low-dose celecoxib did not change the spinal hypersensitivity or movements of animals that underwent the sham operation.** A) Pressure hyperalgesia of the lumbar spine was assessed as the force threshold needed to induce vocalization by a force gauge at 2 and 4 weeks after high-dose or low-dose celecoxib treatment for the mice with the sham operation. B-D) Spontaneous activity analysis, including distance traveled (B), active time (C), and maximum speed (D) on the wheel per 24 h at 2 and 4 weeks after high-dose or low-dose celecoxib treatment for the mice with the sham operation. E, F) The hind paw withdrawal frequency in response to mechanical stimulation (von Frey, 0.07 g and 0.4 g) at 2 and 4 weeks after high-dose or low-dose celecoxib treatment for the mice with the sham operation. **p* < 0.05 compared with the sham group. n = 3 per group.

**Supplementary Figure 2. Celecoxib treatment did not affect the bone mass of the lumbar vertebral body in the mice with LSI surgery.** A) Representative three-dimensional high-resolution μCT images of the trabecular bone of L5 vertebrae (coronal view) at 4 weeks after celecoxib or vehicle treatment. Scale bars, 1 mm. B-E) Quantitative analysis of the trabecular bone volume/total volume (BV/TV, B) and trabecular bone number (Tb.N, C), trabecular bone thickness (Tb.Th, D), and trabecular bone separation distribution (Tb.Sp, E) in L5 vertebrae determined by μCT. **p* < 0.05 compared with the sham group and #*p* < 0.05 compared with the vehicle group at the corresponding time points. n = 6 per group. F) PGE2 levels in the L5 vertebral body and L4-L5 caudal endplates at 2 weeks after celecoxib or vehicle treatment. **p* < 0.05 compared with the vertebral body in the corresponding treatment group and #*p* < 0.05 compared with the sham group in the corresponding tissue (vertebral body or endplate). n = 6 per group.
